# Cancer Variant Interpretation Group UK (CanVIG-UK): an exemplar national subspecialty multidisciplinary network

**DOI:** 10.1136/jmedgenet-2019-106759

**Published:** 2020-03-13

**Authors:** Alice Garrett, Alison Callaway, Miranda Durkie, Cankut Cubuk, Mary Alikian, George J Burghel, Rachel Robinson, Louise Izatt, Sabrina Talukdar, Lucy Side, Treena Cranston, Sheila Palmer-Smith, Diana Baralle, Ian R Berry, James Drummond, Andrew J Wallace, Gail Norbury, Diana M Eccles, Sian Ellard, Fiona Lalloo, D Gareth Evans, Emma Woodward, Marc Tischkowitz, Helen Hanson, Clare Turnbull, CanVIG-UK, Stephen Abbs

**Affiliations:** 1 Division of Genetics and Epidemiology, Institute of Cancer Research, Sutton, UK; 2 Wessex Regional Genetics Laboratory, Salisbury Hospital NHS Foundation Trust, Salisbury, UK; 3 Human Genetics and Genomic Medicin, Faculty of Medicine, University of Southampton, Southampton, UK; 4 Sheffield Diagnostic Genetics Service, Sheffield Children's NHS Foundation Trust, Sheffield, UK; 5 William Harvey Research Institute, Queen Mary University of London, London, UK; 6 Manchester Centre for Genomic Medicine and NW Laboratory Genetics Hub, Manchester University NHS Foundation Trust, Manchester, UK; 7 Yorkshire Regional Genetics Service, Leeds Teaching Hospitals NHS Trust, Leeds, UK; 8 Department of Clinical Genetics, Guy's and Saint Thomas' NHS Foundation Trust, London, UK; 9 Department of Clinical Genetics, Saint George's University Hospitals NHS Foundation Trust, London, UK; 10 Wessex Clinical Genetics Service, Princess Anne Hospital, Southampton, UK; 11 Oxford Molecular Genetics Laboratory, Churchill Hospital, Oxford, UK; 12 Institute of Medical Genetics, University Hospital of Wales, Cardiff, UK; 13 Faculty of Medicine, University of Southampton, Southampton, UK; 14 East Anglian Medical Genetics Service, Cambridge University Hospitals NHS Foundation Trust, Cambridge, UK; 15 Regional Genetics Service, Guy's and Saint Thomas' NHS Foundation Trust, London, UK; 16 Department of Molecular Genetics, Royal Devon and Exeter NHS Foundation Trust, Exeter, UK; 17 Division of Evolution and Genomic Sciences, School of Biological Sciences, Faculty of Biology Medicine and Health, The University of Manchester, Manchester, UK; 18 Department of Medical Genetics, National Institute for Health, Research Cambridge Biomedical Research Centre, University of Cambridge, Cambridge, UK; 19 Cancer Genetics Unit, Royal Marsden NHS Foundation Trust, London, UK

**Keywords:** genetics, clinical genetics, guidelines, molecular genetics, oncology

## Abstract

Advances in technology have led to a massive expansion in the capacity for genomic analysis, with a commensurate fall in costs. The clinical indications for genomic testing have evolved markedly; the volume of clinical sequencing has increased dramatically; and the range of clinical professionals involved in the process has broadened. There is general acceptance that our early dichotomous paradigms of variants being pathogenic–high risk and benign–no risk are overly simplistic. There is increasing recognition that the clinical interpretation of genomic data requires significant expertise in disease–gene-variant associations specific to each disease area. Inaccurate interpretation can lead to clinical mismanagement, inconsistent information within families and misdirection of resources. It is for this reason that ‘national subspecialist multidisciplinary meetings’ (MDMs) for genomic interpretation have been articulated as key for the new NHS Genomic Medicine Service, of which Cancer Variant Interpretation Group UK (CanVIG-UK) is an early exemplar. CanVIG-UK was established in 2017 and now has >100 UK members, including at least one clinical diagnostic scientist and one clinical cancer geneticist from each of the 25 regional molecular genetics laboratories of the UK and Ireland. Through CanVIG-UK, we have established national consensus around variant interpretation for cancer susceptibility genes via monthly national teleconferenced MDMs and collaborative data sharing using a secure online portal. We describe here the activities of CanVIG-UK, including exemplar outputs and feedback from the membership.

## Background

### Clinical utility of cancer susceptibility genes (CSGs)

Analysis of germline (constitutional) variants in CSGs constitutes approximately one-quarter of activity in NHS Molecular Diagnostic Laboratories in England.^1^ Following identification of a pathogenic variant (PV) in a CSG, incidence of/mortality from future cancers may be mitigated via (1) risk-reducing surgery (eg, mastectomy, gastrectomy, salpingo-ophorectomy and colectomy); (2) chemoprevention; (3) intensive screening; and (4) lifestyle modification.^2^ Family members negative for the familial CSG-PV can be spared anxiety and unnecessary screening. Many CSGs are associated with a pattern of cancer risk that is late-onset, variably penetrant and of autosomal dominant inheritance. PV-positive family members identified via cascade screening are often distributed across disparate genomics services.

Erroneous interpretation of CSG variant pathogenicity can therefore result in (1) discordant management within families, (2) serious clinical consequences for individuals and (3) misdirection at population level of resources for screening and prevention.^3–5^ Increasingly, CSG-PVs are used as predictive biomarkers to inform cancer therapy. For all these reasons, robust, rapid, accurate variant analysis and interpretation of disease risk are critical to effective delivery of germline cancer genetics and improving outcomes for patients.

### Evolving landscape of variant interpretation in germline cancer genetics

In the late 1990s, within a few years of identification of the relevant genes, laboratory analysis of CSGs became available in the UK via family cancer clinics.^2^ If the cancer phenotype ascribed to the gene matched that found in the proband/family under study, with little additional evidence, a rare variant would often be labelled as pathogenic and thus causative.^6^ Subsequent large-scale population sequencing studies have revealed the degree of innocuous variation present in the human genome (and indeed in disease-associated genes) and ‘downgrading’ of many erroneously labelled PVs has been required.^7^ An era of caution followed, with much greater recourse to labelling of variants as ‘variants of uncertain significance’ (VUS/VOUS). However, lack of systems for sharing new evidence has meant that many families have spent years in limbo with their ‘VUS’, even when data had long been available by which classification of their variant could be downgraded or upgraded.

Sharing of clinical variant data was somewhat improved with the advent of locus-specific databases (LSDs), such as Breast Cancer Information Core and Leiden Open Variant Databases.^8–11^ However, the curation of clinical and molecular data in LSDs often remains suboptimal, with (1) erroneous nomenclature, (2) duplication of entries and (3) use of differing classification systems resulting in contradictory assignations.^12^

Using Myriad Genetics data from ~70 000 genetic tests for hereditary breast and ovarian cancer, in 2007, Easton and colleagues published a landmark multifactorial analysis through which ‘odds of causality’ were mathematically generated for 1433 variants using clinical, pedigree and allelic data.^13^ In 2008, International Agency for Research on Cancer (IARC) collaborators published the first formal five point variant interpretation system for CSGs, which included numeric thresholds for the probability of pathogenicity.^14^ Expert cancer susceptibility consortia such as the Evidence-based Network for the Interpretation of Germline Mutant Alleles (ENIGMA) and the International Society for Gastrointestinal Hereditary Tumours (InSIGHT) further evolved these multifactorial variant classification systems to incorporate tumour phenotype and in silico predictions.^15–17^ However, ENIGMA/InSIGHT approaches require statistical genetic–epidemiological analyses of large curated data series and are not reproducible by an individual diagnostic laboratory seeking to classify in a clinically relevant timescale a newly identified variant.

In 2015, the American College of Medical Geneticists (ACMG) published a variant interpretation framework enabling the combination by a diagnostic laboratory of disparate evidence sources for a newly identified genomic variant.^18^ The ACMG framework has subsequently been further evolved under the auspices of ClinGen, including (1) specification for how it is applied to particular genes and/or diseases (including *TP53*, *CDH1* and *PTEN*); (2) deeper specification of particular criteria (eg, functional assays); and (3) exposition of the underpinning Bayesian model.^19–23^

### Coordinated national UK approaches in variant interpretation

In 2016, with endorsement from NHS England and Health Education England, it was agreed formally by the UK Association of Clinical Genomic Science (UK-ACGS) to adopt the ACMG variant interpretation framework.^24 25^ The UK-ACGS established national groups for rare disease, germline cancer genetics, cardiac disease and hypercholesterolaemia to develop and disseminate practice in the application of the ACMG variant interpretation framework.^24^ In parallel was recognition within the NHS Genomic Medicine Service of the need for national subspecialist genomics MDMs.^26 27^ In response to these dual recommendations, Cancer Variant Interpretation Group UK (CanVIG-UK) was initiated in 2017.

## Cancer Variant Interpretation Group UK

The purpose of CanVIG-UK is to advance outcomes for patients by improving the accuracy and consistency of interpretation of variants in CSGs across the UK clinical genetics and molecular diagnostic laboratory communities (hereafter termed the UK clinical-laboratory community). We aim to progress this goal by advancing six objectives (see box 1).

Box 1 **CanVIG Objectives**

The purpose of Cancer Variant Interpretation Group UK (CanVIG-UK) is to advance outcomes for patients by improving the accuracy and consistency of interpretation of variants in Cancer Susceptibility genes across the UK clinical-laboratory community. We have six specific objectives:

1. Creation of a national multidisciplinary professional network and regular forum.
2. Training and education.
3. Detailed specification for germline cancer genetics of the UK-ACGS Best Practice Guidelines for Variant Interpretation.
4. Ratification of additional guidance in germline cancer genetics relevant to the UK clinical-laboratory community.
5. Development of an online platform to facilitate information sharing and variant interpretation within the UK clinical-laboratory community.
6. UK contribution to international variant interpretation endeavours.

### Creation of a national multidisciplinary professional network and regular forum

**Figure 1:**
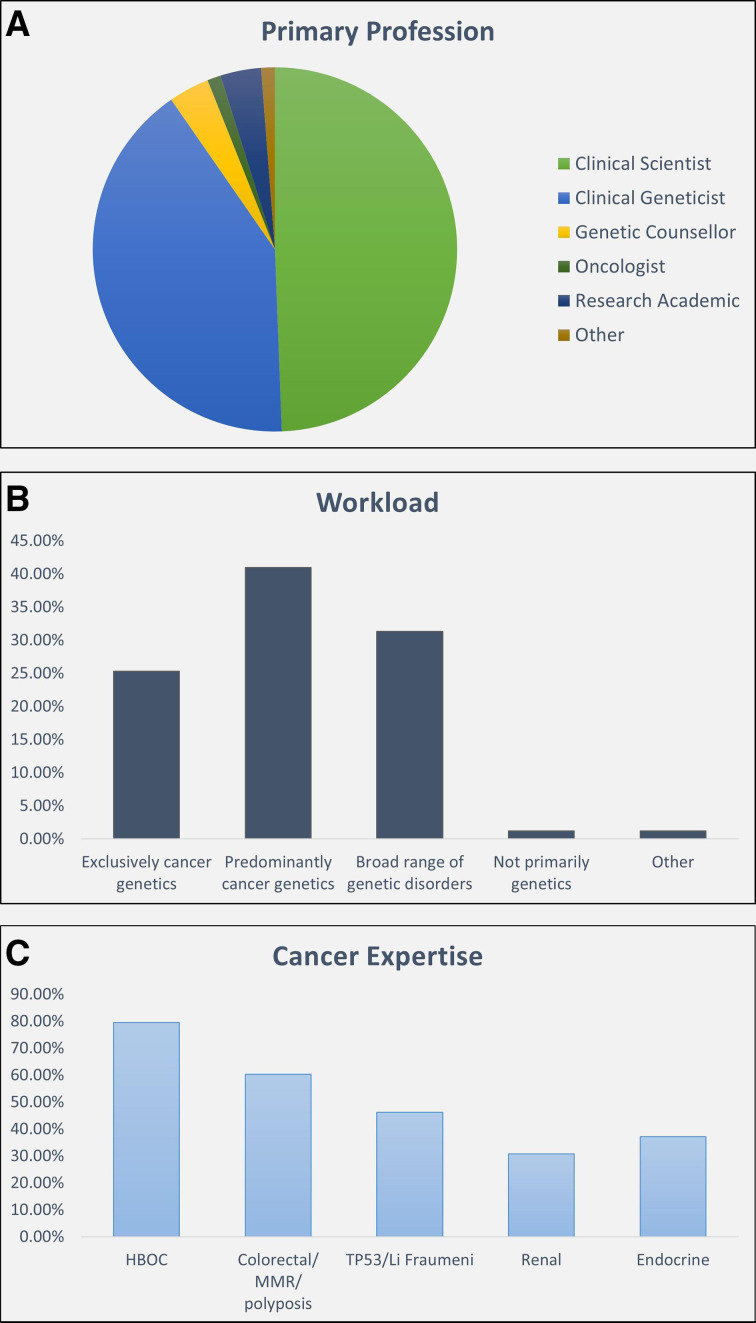
Overview of the CanVIG-UK membership profile (survey of CanVIG-UK members, performed on 29 October 2019, return rate 83/103 (81%). HBOC, hereditary breast and ovarian cancer; MMR, mismatch repair. CanVIG-UK, Cancer Variant Interpretation Group UK.

CanVIG-UK has grown to now include >100 members, incorporating clinical and laboratory representation from each of the 25 Molecular Diagnostic Laboratories and Clinical Genetics Services of the UK (NHS) and Ireland (see collaborators). This group comprises roughly equal proportions of clinical scientists and clinical geneticists, with two-thirds working exclusively or predominantly in cancer genetics (figure 1):

- The monthly teleconferenced MDM provides a *forum* to which problematic variants/cases are submitted. The variants submitted to the monthly variant surgery are circulated 1 week in advance. CanVIG-UK members are asked (1) to ascertain whether additional cases and/or laboratory data exist locally and (2) to undertake local, independent classification of the variant. The relevant clinical and laboratory data are presented by the nominating laboratory. This is followed by input of any additional information by the broader CanVIG-UK group and a discussion regarding the legitimacy of the ACMG criteria awarded. Following this discussion and an online postdiscussion poll, a consensus CanVIG classification is generated (see online supplementary table 1). A detailed date-stamped CanVIG variant summary sheet is generated (see online supplementary appendix 2), which is circulated by email, uploaded to the CanVar-UK portal and submitted to ClinVar.
- The CanVIG-UK *network* is active throughout the month via the email forum, through which urgent queries can be debated and addressed.

10.1136/jmedgenet-2019-106759.supp1 Supplementary data

10.1136/jmedgenet-2019-106759.supp2 Supplementary data

### Training and education

**Figure 2:**
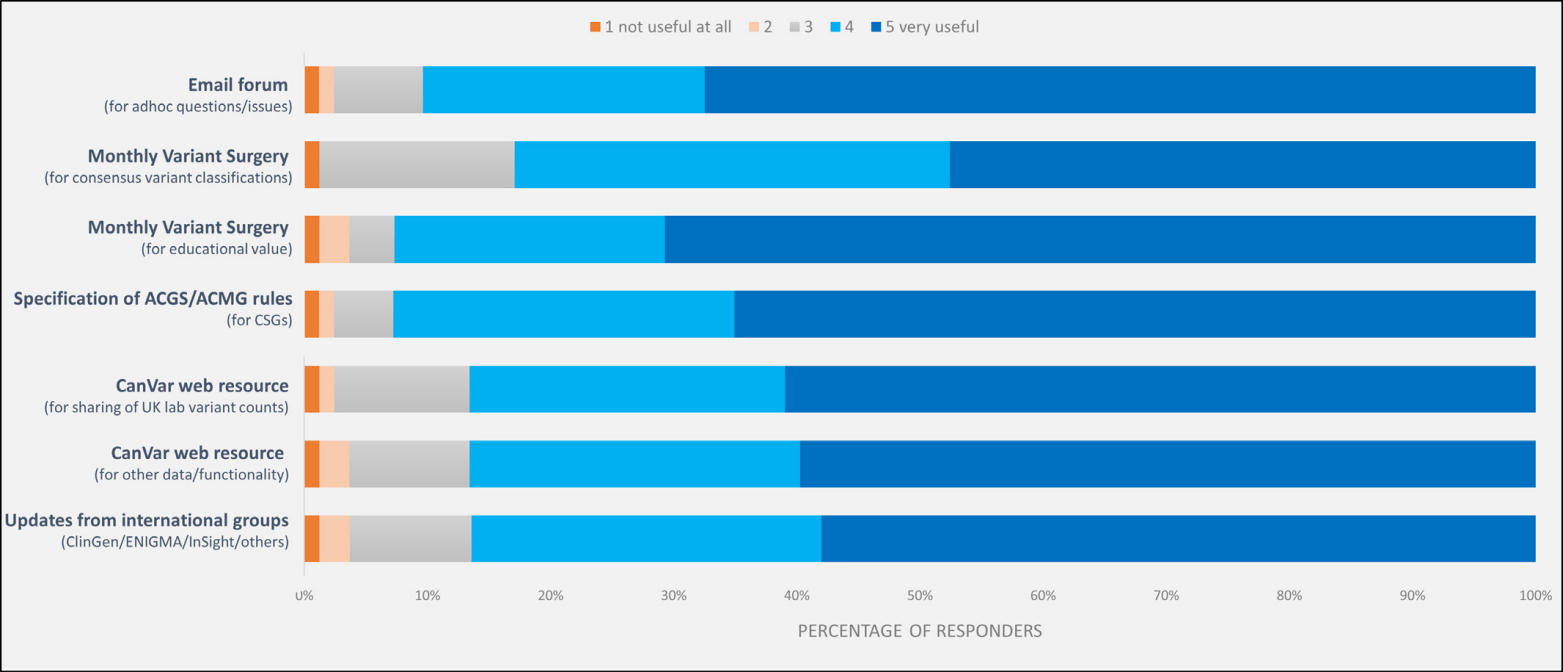
Perceived utility of CanVIG-UK activities regarding local practice in CSG variant interpretation of seven activities (5: very useful to 1: not useful; survey of CanVIG-UK members, performed on 29 October 2019, return rate 83/103 (81%)). ACGS, Association of Clinical Genomic Science; ACMG, American College of Medical Geneticists; CanVIG-UK, Cancer Variant Interpretation Group UK; CSG, cancer susceptibility gene.

The discussion of cases at the MDM also provides valuable education for the clinical-laboratory community regarding application of the ACMG framework and the vagaries of the evidence sources used (see figure 2). Additionally, through CanVIG-UK, we have supported training of the broader UK genetics and oncology communities in variant interpretation for CSGs.

### Detailed specification for germline cancer genetics of the UK-ACGS Best Practice Guidelines for Variant Interpretation

On behalf of the UK-ACGS, the rare disease variant interpretation group has generated and updates annually a highly detailed specification of the ACMG variant interpretation framework.^24^ In cancer susceptibility, we typically observe variants relating to late-onset, common phenotypes. De novo and biallelic paradigms are infrequent. We are typically much more reliant on variant frequency from case series and functional assays. Thus, an important remit for CanVIG-UK has been to develop a detailed specification of the UK-ACGS framework for these types of evidence to be used for CSG variant interpretation (see online supplementary appendix 1).

### Ratification of additional guidance in germline cancer genetics relevant to the UK clinical-laboratory community

Historically, the first presentation to the family cancer clinic was typically an unaffected individual, concerned by a significant family history. Increasingly, genetic analysis is now performed as part of routine work-up at cancer diagnosis, either through analysis of a germline sample or through therapeutically motivated molecular analysis of the tumour. In both contexts, (1) focused testing of one or two genes has often been superseded by broad ‘cancer panels’ containing dozens or hundreds of genes; (2) patients may be unselected for family history; and (3) analysis and reporting in a tight time frame is typically required. A number of challenging issues have emerged, including

1. Categorisation and management of reduced penetrance variants in high-penetrance genes.
2. Variant interpretation and clinical management for moderate-penetrance genes.
3. Adaptation of variant interpretation and risk for different contexts of ascertainment.
4. Inference of germline findings from tumour-only sequencing.

While germane across genomics, consideration of these issues has become pressing within germline cancer genetics. Benefitting from its regular forum, multidisciplinary membership and alignment with both UK-ACGS and the UK Cancer Genetics Group (UK-CGG), we have used the CanVIG–UK monthly forum to evolve UK national multidisciplinary approaches on such issues (see online supplementary appendix 3).

### Development of an online platform to facilitate information sharing and variant interpretation within the UK clinical-laboratory community

In germline cancer genetics, enrichment in cases (especially ‘strong families’) is one of the most valuable clinical observations indicating variant pathogenicity. However, to date, we have struggled to quantify such observations on account of (1) failure to aggregate national data from distributed laboratories and (2) lack of a robust denominator.

In a collaborative venture between Public Health England (PHE) and the national network of molecular diagnostic laboratories, data from molecular testing of CSGs have been submitted via a pseudonymisation portal to the secure National Cancer Registration and Analysis Service (NCRAS) data environment of PHE.^28^ The national variant totals (numerator and denominator) are then shared by CanVIG-UK with the UK clinical-laboratory community via our online data system CanVar-UK (http://www.canvaruk.org/).

CanVar-UK provides additional annotations for 1 008 643 variants from 95 CSGs. It includes variant-level annotations from LSDs (case counts), functional assays, splicing assays and multifactorial analyses for selected genes. Accessible only to registered CanVIG-UK clinical-laboratory users is a community area for sharing non-identifiable variant-level data, such as local classifications, comments/notes, uploaded documents and results from local laboratory assays (eg, RNA analyses of potential splicing variants).

### UK contribution to international variant interpretation endeavours

CanVIG-UK is an effective conduit between the UK clinical-laboratory germline cancer genetics community and relevant international variant interpretation endeavours in several regards:

- First, there is representation at the international ClinGen SVI group from the leadership of the UK-ACGS rare disease variant interpretation group. The regular crosstalk between leadership of the UK groups enables appraisal of the ClinGen SVI group of emerging analyses and activity within CanVIG-UK and the UK clinical-laboratory cancer genetics community.
- Second, multiple members of CanVIG-UK are members of gene-specific international endeavours such as ENIGMA, InSIGHT and ClinGen expert groups.
- Third, data generated by CanVIG-UK data have contributed to collaborative international consortia analyses, for example, provision to ENIGMA of the summary PHE UK laboratory data on BRCA1/BRCA2 variants.
- Fourth, CanVIG-UK consensus classifications (and underpinning evidence) are shared via ClinVar. CanVIG-UK is the first UK organisation to submit clinical-laboratory variant classifications to ClinVar.

### Sustainability

Maintenance of a national multidisciplinary network, coordination of a regular teleconferenced MDMs and development of a data system is only feasible via sustained support. The activities of CanVIG-UK are currently supported by a Cancer Research UK Catalyst Award (CanGene-CanVar, @CangeneCanvar, C61296/A27223).

## Conclusion

CanVIG-UK is a multidisciplinary group comprising >100 clinical scientists and senior genetics clinicians working in germline cancer genetics, with representation from across the 25 NHS molecular diagnostic laboratories of the UK and Ireland. Through CanVIG-UK, the UK clinical-laboratory germline cancer genetics community have evolved:

1. An email forum for real-time consultation on problematic variants.
2. A monthly teleconferenced MDM for detailed review of challenging variants and cases.
3. A national programme of using secure submissions of frequency data from PHE.
4. An online data system (CanVar-UK) for sharing variant-level data both publicly and within a secure community region.
5. Detailed, consensus UK guidance for the interpretation of variants in CSGs.
6. Fruitful interactions with international CSG variant interpretation endeavours.

In summary, we propose CanVIG-UK as an exemplar National Subspecialty Multidisciplinary Genomics Network. In this era of rapid emergence of genomic knowledge, such networks are becoming increasingly important to optimise collaborative specialist case review, information sharing and education.

## References

[1] Norbury G Association of Clinical Genomic Science (ACGS) 2015-2016 Genetic Test Activity Audit 2017.

[2] Turnbull C, Sud A, Houlston RS Cancer genetics, precision prevention and a call to action. Nat Genet 2018;50:1212–8. 10.1038/s41588-018-0202-0 30158684 PMC6420140

[3] Tandy-Connor S, Guiltinan J, Krempely K, LaDuca H, Reineke P, Gutierrez S, Gray P, Tippin Davis B False-Positive results released by direct-to-consumer genetic tests highlight the importance of clinical confirmation testing for appropriate patient care. Genet Med 2018;20:1515–21. 10.1038/gim.2018.38 29565420 PMC6301953

[4] Kilbride MK, Domchek SM, Bradbury AR Ethical implications of direct-to-consumer hereditary cancer tests. JAMA Oncol 2018;4:1327–8. 10.1001/jamaoncol.2018.2439 30027223 PMC6988778

[5] The Guardian, 2019 Available: https://www.theguardian.com/science/2019/jul/21/senior-doctors-call-for-crackdown-on-home-genetic-testing-kits

[6] Eccles DM, Mitchell G, Monteiro ANA, Schmutzler R, Couch FJ, Spurdle AB, Gómez-García EB, ENIGMA Clinical Working Group Brca1 and BRCA2 genetic testing-pitfalls and recommendations for managing variants of uncertain clinical significance. Ann Oncol 2015;26:2057–65. 10.1093/annonc/mdv278 26153499 PMC5006185

[7] Lek M, Karczewski KJ, Minikel EV, Samocha KE, Banks E, Fennell T, O'Donnell-Luria AH, Ware JS, Hill AJ, Cummings BB, Tukiainen T, Birnbaum DP, Kosmicki JA, Duncan LE, Estrada K, Zhao F, Zou J, Pierce-Hoffman E, Berghout J, Cooper DN, Deflaux N, DePristo M, Do R, Flannick J, Fromer M, Gauthier L, Goldstein J, Gupta N, Howrigan D, Kiezun A, Kurki MI, Moonshine AL, Natarajan P, Orozco L, Peloso GM, Poplin R, Rivas MA, Ruano-Rubio V, Rose SA, Ruderfer DM, Shakir K, Stenson PD, Stevens C, Thomas BP, Tiao G, Tusie-Luna MT, Weisburd B, Won H-H, Yu D, Altshuler DM, Ardissino D, Boehnke M, Danesh J, Donnelly S, Elosua R, Florez JC, Gabriel SB, Getz G, Glatt SJ, Hultman CM, Kathiresan S, Laakso M, McCarroll S, McCarthy MI, McGovern D, McPherson R, Neale BM, Palotie A, Purcell SM, Saleheen D, Scharf JM, Sklar P, Sullivan PF, Tuomilehto J, Tsuang MT, Watkins HC, Wilson JG, Daly MJ, MacArthur DG, Exome Aggregation Consortium Analysis of protein-coding genetic variation in 60,706 humans. Nature 2016;536:285–91. 10.1038/nature19057 27535533 PMC5018207

[8] Pinard A, Salgado D, Desvignes J-P, Rai G, Hanna N, Arnaud P, Guien C, Martinez M, Faivre L, Jondeau G, Boileau C, Zaffran S, Béroud C, Collod-Béroud G WES/WGS Reporting of Mutations from Cardiovascular "Actionable" Genes in Clinical Practice: A Key Role for UMD Knowledgebases in the Era of Big Databases. Hum Mutat 2016;37:1308–17. 10.1002/humu.23119 27647783

[9] Pinard A, Miltgen M, Blanchard A, Mathieu H, Desvignes J-P, Salgado D, Fabre A, Arnaud P, Barré L, Krahn M, Grandval P, Olschwang S, Zaffran S, Boileau C, Béroud C, Collod-Béroud G Actionable genes, core databases, and locus-specific databases. Hum Mutat 2016;37:1299–307. 10.1002/humu.23112 27600092

[10] Szabo C, Masiello A, Ryan JF, Brody LC The breast cancer information core: database design, structure, and scope. Hum Mutat 2000;16:123–31. 10.1002/1098-1004(200008)16:2&lt;123::AID-HUMU4&gt;3.0.CO;2-Y 10923033

[11] Fokkema IFAC, Taschner PEM, Schaafsma GCP, Celli J, Laros JFJ, den Dunnen JT LOVD v.2.0: the next generation in gene variant databases. Hum Mutat 2011;32:557–63. 10.1002/humu.21438 21520333

[12] Greenblatt MS, Brody LC, Foulkes WD, Genuardi M, Hofstra RMW, Olivier M, Plon SE, Sijmons RH, Sinilnikova O, Spurdle AB, IARC Unclassified Genetic Variants Working Group Locus-specific databases and recommendations to strengthen their contribution to the classification of variants in cancer susceptibility genes. Hum Mutat 2008;29:1273–81. 10.1002/humu.20889 18951438 PMC3446852

[13] Easton DF, Deffenbaugh AM, Pruss D, Frye C, Wenstrup RJ, Allen-Brady K, Tavtigian SV, Monteiro ANA, Iversen ES, Couch FJ, Goldgar DE A systematic genetic assessment of 1,433 sequence variants of unknown clinical significance in the BRCA1 and BRCA2 breast cancer-predisposition genes. Am J Hum Genet 2007;81:873–83. 10.1086/521032 17924331 PMC2265654

[14] Plon SE, Eccles DM, Easton D, Foulkes WD, Genuardi M, Greenblatt MS, Hogervorst FBL, Hoogerbrugge N, Spurdle AB, Tavtigian SV, IARC Unclassified Genetic Variants Working Group Sequence variant classification and reporting: recommendations for improving the interpretation of cancer susceptibility genetic test results. Hum Mutat 2008;29:1282–91. 10.1002/humu.20880 18951446 PMC3075918

[15] Spurdle AB, Healey S, Devereau A, Hogervorst FBL, Monteiro ANA, Nathanson KL, Radice P, Stoppa-Lyonnet D, Tavtigian S, Wappenschmidt B, Couch FJ, Goldgar DE, ENIGMA ENIGMA-evidence-based network for the interpretation of germline mutant alleles: an international initiative to evaluate risk and clinical significance associated with sequence variation in BRCA1 and BRCA2 genes. Hum Mutat 2012;33:2–7. 10.1002/humu.21628 21990146 PMC3240687

[16] Parsons MT, Tudini E, Li H, Hahnen E, Wappenschmidt B, Feliubadaló L, Aalfs CM, Agata S, Aittomäki K, Alducci E, Alonso-Cerezo MC, Arnold N, Auber B, Austin R, Azzollini J, Balmaña J, Barbieri E, Bartram CR, Blanco A, Blümcke B, Bonache S, Bonanni B, Borg Åke, Bortesi B, Brunet J, Bruzzone C, Bucksch K, Cagnoli G, Caldés T, Caliebe A, Caligo MA, Calvello M, Capone GL, Caputo SM, Carnevali I, Carrasco E, Caux-Moncoutier V, Cavalli P, Cini G, Clarke EM, Concolino P, Cops EJ, Cortesi L, Couch FJ, Darder E, de la Hoya M, Dean M, Debatin I, Del Valle J, Delnatte C, Derive N, Diez O, Ditsch N, Domchek SM, Dutrannoy V, Eccles DM, Ehrencrona H, Enders U, Evans DG, Farra C, Faust U, Felbor U, Feroce I, Fine M, Foulkes WD, Galvao HCR, Gambino G, Gehrig A, Gensini F, Gerdes A-M, Germani A, Giesecke J, Gismondi V, Gómez C, Gómez Garcia EB, González S, Grau E, Grill S, Gross E, Guerrieri-Gonzaga A, Guillaud-Bataille M, Gutiérrez-Enríquez S, Haaf T, Hackmann K, Hansen TVO, Harris M, Hauke J, Heinrich T, Hellebrand H, Herold KN, Honisch E, Horvath J, Houdayer C, Hübbel V, Iglesias S, Izquierdo A, James PA, Janssen LAM, Jeschke U, Kaulfuß S, Keupp K, Kiechle M, Kölbl A, Krieger S, Kruse TA, Kvist A, Lalloo F, Larsen M, Lattimore VL, Lautrup C, Ledig S, Leinert E, Lewis AL, Lim J, Loeffler M, López-Fernández A, Lucci-Cordisco E, Maass N, Manoukian S, Marabelli M, Matricardi L, Meindl A, Michelli RD, Moghadasi S, Moles-Fernández A, Montagna M, Montalban G, Monteiro AN, Montes E, Mori L, Moserle L, Müller CR, Mundhenke C, Naldi N, Nathanson KL, Navarro M, Nevanlinna H, Nichols CB, Niederacher D, Nielsen HR, Ong K-R, Pachter N, Palmero EI, Papi L, Pedersen IS, Peissel B, Perez-Segura P, Pfeifer K, Pineda M, Pohl-Rescigno E, Poplawski NK, Porfirio B, Quante AS, Ramser J, Reis RM, Revillion F, Rhiem K, Riboli B, Ritter J, Rivera D, Rofes P, Rump A, Salinas M, Sánchez de Abajo AM, Schmidt G, Schoenwiese U, Seggewiß J, Solanes A, Steinemann D, Stiller M, Stoppa-Lyonnet D, Sullivan KJ, Susman R, Sutter C, Tavtigian SV, Teo SH, Teulé A, Thomassen M, Tibiletti MG, Tischkowitz M, Tognazzo S, Toland AE, Tornero E, Törngren T, Torres-Esquius S, Toss A, Trainer AH, Tucker KM, van Asperen CJ, van Mackelenbergh MT, Varesco L, Vargas-Parra G, Varon R, Vega A, Velasco Ángela, Vesper A-S, Viel A, Vreeswijk MPG, Wagner SA, Waha A, Walker LC, Walters RJ, Wang-Gohrke S, Weber BHF, Weichert W, Wieland K, Wiesmüller L, Witzel I, Wöckel A, Woodward ER, Zachariae S, Zampiga V, Zeder-Göß C, Lázaro C, De Nicolo A, Radice P, Engel C, Schmutzler RK, Goldgar DE, Spurdle AB, KConFab Investigators Large scale multifactorial likelihood quantitative analysis of BRCA1 and BRCA2 variants: an enigma resource to support clinical variant classification. Hum Mutat 2019;40:1557–78. 10.1002/humu.23818 31131967 PMC6772163

[17] Thompson BA, Spurdle AB, Plazzer J-P, Greenblatt MS, Akagi K, Al-Mulla F, Bapat B, Bernstein I, Capellá G, den Dunnen JT, du Sart D, Fabre A, Farrell MP, Farrington SM, Frayling IM, Frebourg T, Goldgar DE, Heinen CD, Holinski-Feder E, Kohonen-Corish M, Robinson KL, Leung SY, Martins A, Moller P, Morak M, Nystrom M, Peltomaki P, Pineda M, Qi M, Ramesar R, Rasmussen LJ, Royer-Pokora B, Scott RJ, Sijmons R, Tavtigian SV, Tops CM, Weber T, Wijnen J, Woods MO, Macrae F, Genuardi M Application of a 5-tiered scheme for standardized classification of 2,360 unique mismatch repair gene variants in the insight locus-specific database. Nat Genet 2014;46:107–15. 10.1038/ng.2854 24362816 PMC4294709

[18] Richards S, Aziz N, Bale S, Bick D, Das S, Gastier-Foster J, Grody WW, Hegde M, Lyon E, Spector E, Voelkerding K, Rehm HL, ACMG Laboratory Quality Assurance Committee Standards and guidelines for the interpretation of sequence variants: a joint consensus recommendation of the American College of medical genetics and genomics and the association for molecular pathology. Genet Med 2015;17:405–24. 10.1038/gim.2015.30 25741868 PMC4544753

[19] ClinGen variant curation expert panel ClinGen TP53 expert panel specifications to the ACMG/AMP variant interpretation guidelines version 1, 2019 Available: https://clinicalgenome.org/site/assets/files/3876/clingen_tp53_acmg_specifications_v1.pdf2019

[20] Brnich SE, Abou Tayoun AN, Couch FJ, Cutting GR, Greenblatt MS, Heinen CD, Kanavy DM, Luo X, McNulty SM, Starita LM, Tavtigian SV, Wright MW, Harrison SM, Biesecker LG, Berg JS, Clinical Genome Resource Sequence Variant Interpretation Working Group Recommendations for application of the functional evidence PS3/BS3 criterion using the ACMG/AMP sequence variant interpretation framework. Genome Med 2019;12:3 10.1186/s13073-019-0690-2 31892348 PMC6938631

[21] ClinGen variant curation expert panel ClinGen CDH1 expert panel specifications to the ACMG/AMP variant interpretation guidelines version 2, 2019 Available: https://clinicalgenome.org/site/assets/files/3982/clingen_cdh1_acmg_specifications_v2.pdf

[22] Mester JL, Ghosh R, Pesaran T, Huether R, Karam R, Hruska KS, Costa HA, Lachlan K, Ngeow J, Barnholtz-Sloan J, Sesock K, Hernandez F, Zhang L, Milko L, Plon SE, Hegde M, Eng C Gene-Specific criteria for PTEN variant curation: recommendations from the ClinGen PTEN expert panel. Hum Mutat 2018;39:1581–92. 10.1002/humu.23636 30311380 PMC6329583

[23] Tavtigian SV, Greenblatt MS, Harrison SM, Nussbaum RL, Prabhu SA, Boucher KM, Biesecker LG, ClinGen Sequence Variant Interpretation Working Group (ClinGen SVI) Modeling the ACMG/AMP variant classification guidelines as a Bayesian classification framework. Genet Med 2018;20:1054–60. 10.1038/gim.2017.210 29300386 PMC6336098

[24] Ellard S, Baple EL, Berry I, Forrester N, Turnbull C, Owens M, Eccles DM, Abbs S, Scott R, Deans Z, Lester T, Campbell J, Newman W, McMullan D ACGS best practice guidelines for variant classification in rare disease 2020: Association for Clinical Genomic Science (ACGS), 2020 Available: https://www.acgs.uk.com/quality/best-practice-guidelines/#VariantGuidelines

[25] Luheshi L, Hall A, Baple E, Ellard S, McMullan D, Raza, S Variant classication and interpretation workshop report, 2017 Assocation for clinical genomic science (ACGS) and PHG Foundation. Available: https://www.phgfoundation.org/documents/Variant%20classification%20and%20identification%20June%202017.pdf

[26] Turnbull C, Scott RH, Thomas E, Jones L, Murugaesu N, Pretty FB, Halai D, Baple E, Craig C, Hamblin A, Henderson S, Patch C, O'Neill A, Devereau A, Smith K, Martin AR, Sosinsky A, McDonagh EM, Sultana R, Mueller M, Smedley D, Toms A, Dinh L, Fowler T, Bale M, Hubbard T, Rendon A, Hill S, Caulfield MJ, 100 000 Genomes Project The 100 000 Genomes Project: bringing whole genome sequencing to the NHS. BMJ 2018;361:k1687 10.1136/bmj.k1687 29691228

[27] NHS Genomic Medicine Service NHS England, 2019 Available: https://www.england.nhs.uk/genomics/nhs-genomic-med-service/

[28] Cox JH Available: https://www.openpseudonymiser.org/About.aspx

