# Supplementary material for: Cancer Variant Interpretation Group UK (CanVIG-UK): an exemplar national subspecialty multidisciplinary network
Source: J Med Genet. 2020 Mar 13;57(12):829–34. doi: 10.1136/jmedgenet-2019-106759 (PMC7691806; doi:10.1136/jmedgenet-2019-106759)
Supplement: Supplementary data [file jmedgenet-2019-106759supp001.pdf]

Appendix 1: CanVIG-UK Consensus Specification for Cancer Susceptibility Genes of ACGS Best Practice Guidelines for Variant Classification (v1.2 03/03/20)

Guidance notes:

- For the following evidence items, CanVIG-UK have no additional specification for CSGs to add beyond that provided in ACGS Best Practice Guidelines for Variant Classification 2020[1]: PVS1, PS1, PS2, PM4, PM5, PM6, PP1, BS4, BP3, BP7 **(shaded white).**
- For the remaining evidence items, whilst remaining consistent with ACGS Best Practice Guidelines for Variant Classification 2020[1], there are more specific recommendations pertaining to CSGs contained within the CanVIG-UK Consensus Specification (**shaded grey).**
- A number of disease-specific expert panels have been established by the USA ClinGen Sequence Variant Interpretation (SVI) Working Group, generating disease/gene specific variant interpretation guidelines. Following evaluation within the CanVIG-UK group, in subsequent updates to the CanVIG-UK specification, we shall include specific recommendations regarding adoption and implementation of these disease/gene specific-guidance.

**PVS1**	_VSTR	_STR	_MOD	_SUP
Null variant (nonsense, frameshift, canonical ±1 or 2 splice sites, initiation codon, single or multi-exon deletion) in a gene where LOF is a known mechanism of disease				
See LOF decision tree and criteria (Tayoun *et al* 2018)[2]				

**PS1**	_STR	_MOD	_SUP
Same amino acid change as a previously established pathogenic variant, regardless of nucleotide change			
Use at Strong for a missense variant under evaluation whereby there is a reference missense variant classified as (likely) pathogenic that results in the same amino acid change<br>Use at Moderate for an initiation codon variant under evaluation whereby there is a reference variant in the initiation codon classified as (likely) pathogenic<br>Use at Supporting for a donor/acceptor splice region variant under evaluation whereby there is a reference variant at the same base residue classified as (likely) pathogenic. The variant under evaluation must be predicted on *in silico* tools to be equally or more deleterious than the reference variant			

**PS2, PM6**	_VSTR	_STR	_MOD	_SUP
PS2*: De novo (both maternity and paternity confirmed) in a patient with the disease and no family history*<br>***PM6:*** *Assumed de novo, but without confirmation of paternity and maternity*				
See ClinGen Sequence Variant Interpretation Recommendation for *de novo* Criteria (PS2/PM6)[3] https://www.clinicalgenome.org/site/assets/files/3461/svi_proposal_for_de_novo_criteria_v1_0.pdf).				

**PS3**	_VSTR	_STR	_MOD	_SUP
Well-established in vitro or in vivo functional studies supportive of a damaging effect on the gene or gene product				
For assays of protein function				

	Discrimination	Controls	Reproducibility
Strong	relative protein activity assay or functional impact <25% compared to level for wildtype	≥10 'true positive' ≥10'true-negative'	≥2 laboratories OR results demonstrably reproducible from a single laboratory
Moderate		≥5 'true positive' ≥5 'true-negative'	
Supporting		≥2 'true positive' ≥2 'true-negative'	single laboratory

For assays of splicing function

Garrett A, *et al. J Med Genet* 2020; 57:829–834. doi: 10.1136/jmedgenet-2019-106759

Very strong	**2 orthogonal assays:** exhibiting abnormal transcripts; no evidence of leakiness (see notes 4,8)
Strong	**1 assay:** exhibiting abnormal transcripts; no evidence of leakiness (see note 8)
Moderate	**≥1 assay:** exhibiting abnormal transcripts; evidence of leakiness (see note 8)
Supporting	**≥1 assay:** exhibiting abnormal/alternative transcripts which have been reported as present in normal controls (implying naturally occurring isoforms) (see note 12)
**Do not apply**	**≥1 assay:** exhibiting abnormal/alternative transcripts with evidence of extreme leakiness (see note 8)

Explanatory Notes (all functional assays):

- This criterion is for variant-specific analyses. Where functional data provides support at the gene rather than variant level (e.g. biochemical analysis), this should typically be incorporated within the phenotypic specificity criterion PP4
- To be adopted by CanVIG-UK, a published assay requires independent review by two CanVIG-UK registered clinical laboratory scientists
- Guidance for more quantitative evaluation of functional assays has recently been published by the Clinical Genome Resource Sequence Variant Interpretation Working Group (Brnich *et al* 2020)[4]. Evaluation of these approaches by CanVIG-UK is underway but additional training is required. We anticipate adoption as best practice of these more quantitative approaches for review of functional assays and will use the pan-CanVIG network to collate 'ratings' per assay

Explanatory Notes (assays of splicing function):

1. Experimental data may include quantitative assays (e.g. realtime-PCR, Sanger sequencing with formal quantitation of peak height, tape-station quantification of PCR products, minigene assay, RNAseq using NGS) and semi/non-quantitative assays (e.g. visual evaluation of the relative peak height of Sanger sequencing, gel-based evaluation and visualisation of reverse transcriptase PCR (RT-PCR) products, or analysis for evidence of nonsense mediated decay (e.g. where a SNV in trans with the putative splicing variant appears homozygous on RNA sequencing despite being heterozygous on DNA sequencing, indicating the loss of expression of the transcript containing the putative splicing variant))
2. Laboratory methodology should be appropriately validated: primers must have been tested in ≥5 independent normal control reactions, not necessarily run at the same time (i.e. primers could be validated using 5 normal controls across several runs or runs as a batch on a single run)
3. Assays must be performed in a diagnostically ISO accredited laboratory or recognized research laboratory with which direct consultation can be undertaken. **If evidence is derived from an alternative source (e.g. publication only), downgrade by one level of evidence.** All assays should evidence appropriate validations and controls (see note 2).
4. Combinations of assays deemed orthogonal include (a) two PCR-based assays using different primers (b) ≥2 different platforms e.g. RT-PCR and minigene
5. To attain very strong/strong, the criteria by which the disease mechanism is interpreted as loss of function should be met (as per PVS1 recommendations, Tayoun *et al* (2018)[2])
6. The exon in question must be present in the biologically relevant transcript
7. Splicing impact must fulfil one of the criteria below, **otherwise downgrade by one level of evidence**
    a) out of frame + predicted to undergo NMD + removal of >10% of the protein
    b) in-frame but removal of a critical hotspot (as listed in PM1)
    c) in-frame but removal of >10% of the protein
8. Although there will inevitably be gene by gene and exon by exon variation regarding the lower limit of % normal transcripts ('leakiness') at which normal protein function is maintained, this information is not always known. In the absence of specific data for a given gene/exon, the following thresholds of 'leakiness' should be applied:
    - **Evidence against leakiness**: ratio for allele of >80:20 (abnormal: normal) ==overall ratio of >40:60 (abnormal: normal)
    - **Evidence of some leakiness:** ratio for allele of >20:80 (abnormal: normal) ==overall ratio of >10:90 (abnormal: normal)
    - **Evidence of extreme leakiness:** ratio for allele of <20:80 (abnormal: normal) ==overall ratio of < 10:90 (abnormal: normal). Typically, abnormal transcript will be visible on gel but present only at extremely low level or not visible by Sanger sequencing

    The accuracy of different assays in correctly quantifying ratios of different transcripts will vary and is often poorly quantified. As improved data on the precision of different assays emerges, these

Garrett A, *et al. J Med Genet* 2020; 57:829–834. doi: 10.1136/jmedgenet-2019-106759

standards will likely be amended
9. For ±1 or ±2, PVS1 criteria should be used instead of PS3
10. When PS3 is applied for splicing, PP3 (*in silico* evidence), PM4 (in-frame aberration) and PVS1 (truncating) cannot be applied
11. Although PP3 cannot be applied alongside PS3, the assay results for variants at the intron-exon boundaries should nevertheless be supported by *in silico* predictions (MaxEntScan ≥15% difference **OR** SSFL ≥5% difference), **otherwise downgrade by one level of evidence.** Exceptions where *in silico* concordance is not required: (i) U12 splice sites, (ii) TCCTTAAC at the 3' end, (iii) variants outside of intron-exon boundaries (namely 5': Last 3 bases of exon plus 8 bases on intron 3':12 bases of intron plus 2 bases of exon)
12. Naturally occurring (i.e. non-pathogenic) splice variants have been catalogued by expert groups (for example ENIGMA at https://enigmaconsortium.org/library/general-documents/enigma-classification-criteria/)[5] and in reference transcriptome resources (for example GTEX, https://gtexportal.org/home/)[6]

PS4 _VSTR _STR _MOD _SUP

The prevalence of the variant in affected individuals is significantly increased compared with the prevalence in controls

**Vstrong**	$P_{exact} \leq 0.0025$
**Strong**	$P_{exact} \leq 0.05$
**Mod**	$P_{exact} \leq 0.1$
**Sup**	$P_{exact} \leq 0.2$

	Cases	Controls	Total
Variant	a	b	a+b
WT	c	d	c+d
Total	a+c	b+d	a+b+c+d

Explanatory Notes:
- The P_{exact} is generated from Fishers exact 2-way case control comparison (ad/bc)
- Non-duplicated, robustly genotyped case data and control data from equivalent ethic groups are required
- For Western European case data, comparison to the gnomAD NFE population is recommended (i.e. 64,603 NFE individuals for gnomAD v2.1)
- Where there is no count for the variant in gnomAD v2.1, it is not currently possible to ascertain the correct frequency for the wildtype allele. It is recommended that frequency is inferred from inspection at a nearby base at which a variant has been called. If this is not possible, we recommend using a denominator of 90% of the population size (ie 90% x 64,603 NFE individuals, ie 58,143 individuals) to approximate for the frequency at that base accounting for failed calls
- The p-value does not reflect effect size. Therefore, the Odds Ratio (OR) from this case control comparison (ad/bc) should be consistent with the effect size anticipated for that gene type
    - For a 'high penetrance' gene or variant, OR >4 for unselected cancer series or OR>8 for enriched familial cases
    - For an 'intermediate penetrance' gene or reduced penetrance variant in high penetrance gene, OR >2 for unselected cancer series or OR>4 for enriched familial cases
- If the control frequency is 0, the Haldane-Anscombe correction is required to generate an OR (add 0.5 to cells a,b,c,d)
- If there is uncertainty regarding duplicates in the case series, a commensurately more stringent p-value should be applied
- For non-coding variants, restriction to the WGS partition of gnomAD is required
- Where paired numerator-denominator frequencies are unavailable, a case-counting approach is required, which takes into account the specificity of phenotype observed in the proband +/- family. This approach does not take into account the denominator of the reference case series. For *TP53* and *PTEN*, case-counting guidance has been issued via the respective ClinGen expert groups[7] [8]. For MMR, a case-counting approach is under development by CanVIG-UK
- For other rare syndrome cancer susceptibility genes, the UK-ACGS rare disease guidance can be applied. Namely: PS4 can be used at a moderate level of evidence if the variant has been previously identified in multiple (two or more) unrelated affected probands/families with a pathognomonic spectrum of disease, or at a supporting level of evidence if previously identified

Garrett A, *et al. J Med Genet* 2020; 57:829–834. doi: 10.1136/jmedgenet-2019-106759

in one unrelated affected individual, and has not been reported in gnomAD (in a matched ethnic group)

**PM1, PP2** _MOD _SUP
PM1:** Located in a mutational hot spot and/or critical and well-established functional domain (e.g. active site of an enzyme) without benign variation*<br>PP2:** Missense variant in a gene that has a low rate of benign missense variation and in which missense variants are a common mechanism of disease*
Use PM1 for missense variants arising in a CSG domain well characterised as a "hotspot" for pathogenic missense variants<br>Use PM1 at Moderate for a variant in a mutational hotspot at which there is no benign variation<br>Use PM1 at Supporting for a variant in a mutational hotspot at which there is some benign variation<br>Use PP2 at Supporting where there is overall constraint for missense variation at the level of the region/exon/gene (Z≥3.09)<br>**Explanatory Notes:**<br>• The majority of CSGs act by loss of function. Hence, for many of these genes, the majority of established pathogenic variants are truncating (early linkage analyses, agnostic to mechanism, support this). Examples: *BRCA1, BRCA2, PALB2, RAD51C, RAD51D, MLH1, MSH2, MSH6, PMS2.* However, in these genes, there are typically specific domains in which missense variation at key residues can cause loss of function. Where benign variation typically also occur in these regions, PM1_sup can be used. e.g. residues listed in by ENIGMA within *BRCA1* (BRCT and RING domains) and *BRCA2* (DNA binding domain) (https://enigmaconsortium.org/wp-content/uploads/2018/10/ENIGMA_Rules_2017-06-29-v2.5.1.pdf) [5]

**PM2** _MOD _SUP
Absent from controls (or at extremely low frequency if recessive) in Exome Sequencing Project, 1000 Genomes Project, or Exome Aggregation Consortium
Use at Moderate: where 0 observations of the variant in control series >50,000 individuals<br>Use at Supporting where 1 observation on the variant in control series >50,000 individuals<br>**Explanatory Notes:**<br>● Ensure that sequencing coverage is sufficient<br>● Be cautious in using this criterion for small insertions/deletions, as sequencing approaches/analytical methodologies can result in wide variation in calling of these variant types in NGS/exome/genome data<br>● If PS4 has been used for case control data<br>○ The same dataset cannot be re-used for PM2<br>○ An alternative dataset may be used for PM2 of >50,000 individuals of the same ethnicity as that applied for PS4 **OR**<br>○ An alternative dataset may be used for PM2 of >50,000 individuals of ethnicity(ies) different to those for PS4, e.g. If the gnomAD NFE has been used for PS4, the remainder of the GNOMAD populations may be used for PM2 (eg 76,853 individuals non NFE from gnomAD v2.1, if NFE used for PS4)<br>● If PS4 case control data has NOT been used<br>○ A dataset >50,000 individuals of the <u>same</u> ethnicity as your reference case/family **must** be used for PM2

**PM3** _STR _MOD _SUP
For recessive disorders, detected in trans with a pathogenic variant
Use where variant found in trans with a pathogenic variant and the patient-level clinical features match those anticipated for the gene in question<br>Use at **Strong** where variant found in ≥2 unrelated cases, and the features are distinctive for that gene<br>Use at Moderate where variant found in 1 case, and the features are distinctive for that gene<br>Use at Supporting where variant found in 1 case, and the features are distinctive for a set of genes<br>**Explanatory Notes:**

Garrett A, *et al. J Med Genet* 2020; 57:829–834. doi: 10.1136/jmedgenet-2019-106759

• Comprehensive analysis should be undertaken for the gene to exclude an alternative second pathogenic variant (e.g. including MLPA) in that gene<br>• Comprehensive analysis should be undertaken for all other genes for which the phenotypic features overlap<br>• Requires testing of parents (or offspring) to confirm phase<br>• Can use for homozygous variants but downgrade by one evidence level (as per ClinGen SVI points-based system)[9]

**PM4**	_MOD	_SUP
Protein length changes as a result of in-frame deletions/insertions in a non-repeat region or stop-loss variants		
Use at Moderate for<br>• In-frame insertions/deletions for which PVS1 is not applicable (Tayoun et al 2018[2])<br>Use at Supporting for<br>• In-frame insertions/deletions of a single amino-acid		

**PM5**	_MOD	_SUP
Novel missense change at an amino acid residue where a different missense change determined to be pathogenic has been seen before		
Use at Moderate if<br>• Reference variant is classified as pathogenic [OR likely pathogenic and reported in >1 individual]<br>Use at Supporting if<br>• Reference variant is classified likely pathogenic and only reported in 1 individual		

PM6: see above (PS2)

**PP1**	_STR	_MOD	_SUP
Co-segregation with disease in multiple affected family members in a gene definitively known to cause the disease			
• See Jarvik and Browning (2016)[10]			

PP2: see above (PM1)

**PP3**	_SUP
Multiple lines of computational evidence support a deleterious effect on the gene or gene product (conservation, evolutionary, splicing impact)	
• Protein impact: Using a ***predefined*** strategy of<br>○ 3/3 tools (one tool may be marginally below threshold)<br>■ SIFT (deleterious), Polyphen HumVar ≥ (probably damaging) plus:<br>• Align GVGD (C45, C65), (for *BRCA1, BRCA2*) **OR**<br>• MAPP (bad) (for MMR genes) **OR**<br>• CADD (>15) (for any other CSG)<br>○ Or use Revel (>0.7) as a single score<br>• Splicing impact:<br>○ Intron-exon boundary: MaxEnt >15% difference **AND** SSFL >5% difference[11]<br>○ Deep intronic: predicted creation of a novel splice site of any strength, absent in the normal sequence	

Garrett A, *et al. J Med Genet* 2020; 57:829–834. doi: 10.1136/jmedgenet-2019-106759

PP4 _STR _MOD _SUP

Patient's phenotype or family history is highly specific for a disease with a single genetic aetiology

Level	Points	Cellular/molecular phenotype	Example
-	0.5	Moderately predictive for germline aberration of one of a small set of genes	MSI (for mismatch repair deficiency)
Sup	1	Highly predictive for germline aberration of one of a small set of genes	Aberration on mitomycin-induced chromosomal breakage (for genes related to Fanconi Anaemia)
Sup	1	Moderately predictive for germline aberration of the specific gene	LOH at chromosomal locus of tumour-suppressor gene
			Loss on immunohistochemistry of single protein eg MSH6, PMS2
Mod	2	Highly predictive for germline aberration of the specific gene	Depletion of BRCA2 in lymphocytes **and** aberration on mitomycin-induced chromosomal breakage (for *BRCA2*-related Fanconi Anaemia)
			Loss on immunohistochemistry of paired mismatch repair proteins e.g. *MSH2* **and** *MSH6*
			Loss of *MLH1+PMS2* on immunohistochemistry **and** normal *MLH1* promoter methylation (for *MLH1*-related mismatch repair deficiency)

Explanatory Notes:

- For CSGs, PP4 is largely applied for a cellular/molecular phenotype that implicates a particular gene or gene-set
- Comprehensive analysis of the gene and related genes should have been undertaken to exclude an alternative pathogenic variant (including MLPA)
- Individuals/tumours included must have been demonstrated to carry the germline variant
- Evidence can be summed across multiple cases:
  - Total points: Suporting:1; Moderate: 2; Strong: 4
  - Only one individual per family can contribute
- Up to two ***independent*** tumour phenotype assays can be included per case (e.g. MSI AND LOH). Strongly correlated tumour phenotypes from the same case cannot both be included, e.g. MSI and IHC
- For CSGs the high-level clinical phenotype is often too non-specific (e.g. breast and/or ovarian cancer). For a number of pleiomorphic rare tumour and/or syndromic presentations of cancer susceptibility, the specificity of high level clinical phenotype has been captured within PS4 within the case counting guidance (e.g. ClinGen criteria for *CDH1, PTEN, TP53*[7 8 12]). For other pleiomorphic rare tumour and/or syndromic presentations (e.g. MEN1, HLRCC), such specifications are awaited. If molecular data have been used within PS4 as part of a case counting exercise, then PP4 should not be applied

PP5 _SUP

Reputable source recently reports variant as pathogenic, but the evidence is not available to the laboratory to perform an independent evaluation

- Any classification of LP/P after 2016 from
  - ≥2 accredited North American diagnostic laboratories OR
  - a single North American diagnostic laboratory where the utilised evidence is clearly cited
  - an approved ClinGen Expert Group (3 star on ClinVar), e.g. INSIGHT, ENIGMA
- When a single laboratory has classified as LP/P with provision of insufficient detail, it is advised that the individual laboratory is contacted to procure directly the evidence used for classification

Garrett A, *et al. J Med Genet* 2020; 57:829–834. doi: 10.1136/jmedgenet-2019-106759

Additional comments:

- This is an **exceptional** application, as per UK-ACGS specification. For widely tested cancer susceptibility genes, classifications by large laboratories may have been derived from their substantial series of case data not otherwise publicly available

BA1/BS1 _SA _STR

Allele frequency is "too high" for disorder (ExAC or GnomAD)

Use **BA1** as **Stand_Alone** when allele frequency in a large dataset of heterogeneous outbred population (>10,000 individuals) is: >1% or >0.5% (*BRCA1, BRCA2, MLH1, MSH2*)
Use **BS1** as **Strong** when allele frequency in a heterogeneous outbred population is > value specified for specific gene by respective expert group

Explanatory Notes:

- Occasional pathogenic founder mutations occurring at an appreciable frequency in Western Europeans were identified in early characterisation of autosomal dominant CSGs (e.g. *CHEK2* 1100delC at ~0.6%). However, large volumes of sequencing in Western Europeans have now been performed for routinely tested CSGs. The reduction of the threshold of BA1 from 5% to 1% for CSGs and to 0.5% for very well characterised CGSs is predicated on existence of sufficiently high volumes of sequencing data to preclude the existence of hitherto undescribed common founder mutations

BS2/BP2 _STR _SUP

BS2: *Observation in controls inconsistent with disease penetrance. Observed in a healthy adult individual for a* ***recessive (homozygous),*** *dominant (heterozygous), or X-linked (hemizygous) disorder, with full penetrance expected at an early age*
BP2: *Observed* ***in trans*** *with a pathogenic variant for a fully penetrant dominant gene/disorder or observed in cis with a pathogenic variant in any inheritance pattern*

Use BP2 or BS2 at **Supporting** where no further genotyping or clinical/cellular phenotyping is possible
Use BS2 at **Strong** where

- laboratory analysis has been repeated using an orthogonal approach (e.g. different primers) to confirm homozygosity for allele AND
- patient is of age at which biallelic pathogenic variants would be anticipated to be penetrant for a distinctive phenotype AND
- patient has been actively examined to exclude relevant phenotype AND/OR had analysis of cellular phenotype

OR the homozygote is observed in a specified control population in addition to a heterozygote frequency meeting BS1
Use BP2 at **Strong** where

- alleles have been confirmed as in trans AND
- patient is of age at which biallelic pathogenic variants would be anticipated to be penetrant for a distinctive phenotype AND
- patient has been actively examined to exclude relevant phenotype AND/OR had analysis of cellular phenotype

Explanatory Notes:

- BS2 should only be used in the recessive context and for observation of a **homozygote**
- BP2 is used for where the variant is reported as a **compound heterozygote** in conjunction with a pathogenic variant in unaffected individual
- For cancer susceptibility genes, **BP2 and BS2** should only be used for those genes in which typical (non-hypomorphic) biallelic variants cause a recognised phenotype that is fully penetrant from infancy. Such genes include *BRCA2, PALB2, MLH1, MSH2, MSH6, PMS2*

Garrett A, *et al. J Med Genet* 2020; 57:829–834. doi: 10.1136/jmedgenet-2019-106759

BS3 _STR _SUP

Well-established in vitro or in vivo functional studies show no damaging effect on protein function or splicing

For assays of protein function

	Discrimination	Controls	Reproducibility
**Strong**	relative protein activity assay or functional impact	≥10 'true positive' ≥10 'true-negative'	≥2 laboratories OR Results demonstrated as reproducible in single laboratory
**Sup**	>25% compared to level for wildtype	≥2 'true positive' ≥2 'true-negative'	single laboratory

For assays of splicing function

**Strong**	**1 assay:** with no evidence of abnormal transcripts (% normal transcript>90%)	ISO accredited laboratory or recognized research laboratory with which direct consultation can be undertaken
**Sup**	**1 assay:** with no evidence of abnormal transcripts (% normal transcript>90%)	alternative source of evidence (e.g. publication)

Explanatory Notes:

- BS3 should only be applied for an assay of protein function whereby the assay has been validated for variants in the relevant domain to ensure that the mechanism of pathogenicity captured by the assay in question is relevant to that variant
- BS3 should not be applied for an assay of protein function when *in silico* tools predict effect on splicing and/or for the first or last three bases of the exon
- A splicing assay can only be used for BS3 for intronic variants and those in the first or last three bases of the exon
- Evidence of amplification of both alleles is required (i.e. sequencing should demonstrate the SNV in question or another nearby SNV, on the background of the wildtype sequence). This is necessary to exclude generation of a 'normal' RNA result when the splicing aberration has not been detected by the assay used (e.g. due to intron retention, size too large for the PCR to amplify)
- When BS3 is applied for splicing, BP4 (*in silico* evidence), **cannot** be applied. For assays of protein function BS3 and BP4 can be combined.
- For specification of acceptable assays and QC standards, see PS3

BS4 _STR

Non segregation with disease

- See Jarvik and Browning (2016)[10]
- For cancer susceptibility genes for which the phenotype is non-specific and/or feature age-related/reduced penetrance, phenocopies or hypomorphic variants, expert review is recommended for application of BS4 pertaining to non-segregation.

BP1 _SUP

Missense variant in a gene for which primarily truncating variants are known to cause disease

Use at **Supporting** for genes/gene regions in which >95% of reported pathogenic variants are truncating

Explanatory note:

- Can be used outside of BRCT and RING domains for *BRCA1* and outside of DNA-binding domain for *BRCA2.* Other examples of genes for which criterion can be used include *PALB2* and *APC*
- Variant should be evaluated to exclude splicing impact
- Should not be used if the specific amino acid residue is highly conserved in mammals

BP2: see above (BS2)

Garrett A, *et al. J Med Genet* 2020; 57:829–834. doi: 10.1136/jmedgenet-2019-106759

**BP3**	_SUP
In-frame deletions in a repetitive region without a known function	
• Particularly relevant to poorly conserved regions	

**BP4**	_SUP
Multiple lines of computational evidence suggest no impact on gene or gene product (conservation, evolutionary, splicing impact, etc.)	
● Splicing impact:<br>○ Intron-exon boundary: Minimal difference in readings for each of MaxEnt AND SSFL<br>○ AND no evidence of prediction of exonic/deep intronic novel splice site of any strength<br>**AND**<br>● Protein impact: Using a ***predefined*** strategy of<br>○ 3/3 tools (one tool may be marginally above threshold)<br>■ SIFT (tolerated), Polyphen HumVar (benign) plus:<br>● Align GVGD (C0, C15), (for BRCA1, BRCA2)<br>● MAPP (good) (for MMR genes)<br>● CADD (<10) (for any other CSG)<br>○ Or Revel (<0.4) as a single score	

**BP5**	_SUP
Variant found in a case with an alternate molecular basis for disease	
**This should not be applied** for autosomal dominant incompletely penetrant non-syndromic genes associated with common cancers e.g. HBOC (hereditary breast and ovarian cancer)<br>**Explanatory note:**<br>● Co-occurrence of ≥2 pathogenic variants in different cancer susceptibility genes is widely reported. Typically, the phenotype exhibited is indistinguishable from that of a single pathogenic variant	

**BP6**	_SUP
Reputable source recently reports variant as benign, but the evidence is not available to the laboratory to perform an independent evaluation	
**Explanatory Notes:**<br>● Any classification of LB/B after 2016 from<br>○ ≥2 accredited North American diagnostic laboratory OR<br>○ a single North American diagnostic laboratory where the utilised evidence is clearly listed<br>○ ClinGen Expert Group, e.g. INSIGHT, ENIGMA<br>● When a single laboratory has classified as LB/B with provision of insufficient detail, it is advised that the individual laboratory is contacted to procure directly the evidence used for classification	
**Additional comments:**<br>This is an **exceptional** application, as per UK-ACGS specification, as for commonly tested cancer susceptibility genes, classifications by large laboratories may have be derived from their substantial series of case data not otherwise publicly available	

**BP7**	_SUP
A synonymous (silent) variant for which splicing prediction algorithms predict no impact to the splice consensus sequence nor the creation of a new splice site AND the nucleotide is not highly conserved	
• Not to be used if any cause for suspicion of an impact on splicing	

References

Garrett A, *et al. J Med Genet* 2020; 57:829–834. doi: 10.1136/jmedgenet-2019-106759

1. Ellard S, Baple EL, Berry I, Forrester N, Turnbull C, Owens M, Eccles DM, Abbs S, Scott R, Deans Z, Lester T, Campbell J, Newman W, McMullan D. ACGS Best Practice Guidelines for Variant Classification 2020: Association for Clinical Genetics Science (ACGS), 2020.
2. Abou Tayoun AN, Pesaran T, DiStefano MT, Oza A, Rehm HL, Biesecker LG, Harrison SM. Recommendations for interpreting the loss of function PVS1 ACMG/AMP variant criterion. *Human mutation* 2018;39(11):1517-24. doi: 10.1002/humu.23626 [published Online First: 2018/09/08]
3. ClinGen. ClinGen Sequence Variant Interpretation Recommendation for de novo Criteria (PS2/PM6) version 1.0-, 2018.
4. Brnich SE, Abou Tayoun AN, Couch FJ, Cutting GR, Greenblatt MS, Heinen CD, Kanavy DM, Luo X, McNulty SM, Starita LM, Tavtigian SV, Wright MW, Harrison SM, Biesecker LG, Berg JS. Recommendations for application of the functional evidence PS3/BS3 criterion using the ACMG/AMP sequence variant interpretation framework. *Genome medicine* 2019;12(1):3. doi: 10.1186/s13073-019-0690-2 [published Online First: 2020/01/02]
5. Spurdle A. ENIGMA BRCA1/2 Gene Variant Classification Criteria 2017 [Available from: https://enigmaconsortium.org/wp-content/uploads/2018/10/ENIGMA_Rules_2017-06-29-v2.5.1.pdf.
6. The Genotype-Tissue Expression (GTEx) project. *Nature genetics* 2013;45(6):580-5. doi: 10.1038/ng.2653 [published Online First: 2013/05/30]
7. Mester JL, Ghosh R, Pesaran T, Huether R, Karam R, Hruska KS, Costa HA, Lachlan K, Ngeow J, Barnholtz-Sloan J, Sesock K, Hernandez F, Zhang L, Milko L, Plon SE, Hegde M, Eng C. Gene-specific criteria for PTEN variant curation: Recommendations from the ClinGen PTEN Expert Panel. *Human mutation* 2018;39(11):1581-92. doi: 10.1002/humu.23636 [published Online First: 2018/10/13]
8. Savage SA. TP53 Rule Specifications for the ACMG/AMP Variant Curation Guidelines clinicalgenome.org: ClinGen; 2019 [Available from: https://clinicalgenome.org/site/assets/files/3876/clingen_tp53_acmg_specifications_v1.pdf2019.
9. ClinGen. SVI Recommendation for in trans Criterion (PM3) - Version 1.0, 2019.
10. Jarvik GP, Browning BL. Consideration of Cosegregation in the Pathogenicity Classification of Genomic Variants. *American journal of human genetics* 2016;98(6):1077-81. doi: 10.1016/j.ajhg.2016.04.003 [published Online First: 2016/05/31]
11. Houdayer C, Caux-Moncoutier V, Krieger S, Barrois M, Bonnet F, Bourdon V, Bronner M, Buisson M, Coulet F, Gaildrat P, Lefol C, Leone M, Mazoyer S, Muller D, Remenieras A, Revillion F, Rouleau E, Sokolowska J, Vert JP, Lidereau R, Soubrier F, Sobol H, Sevenet N, Bressac-de Paillerets B, Hardouin A, Tosi M, Sinilnikova OM, Stoppa-Lyonnet D. Guidelines for splicing analysis in molecular diagnosis derived from a set of 327 combined in silico/in vitro studies on BRCA1 and BRCA2 variants. *Human mutation* 2012;33(8):1228-38. doi: 10.1002/humu.22101 [published Online First: 2012/04/17]
12. ClinGen CDH1 Expert Panel Specifications to the ACMG/AMP Variant Interpretation Guidelines Version 2, 2019.
