# Supplementary material for: Cancer Variant Interpretation Group UK (CanVIG-UK): an exemplar national subspecialty multidisciplinary network
Source: J Med Genet. 2020 Mar 13;57(12):829–34. doi: 10.1136/jmedgenet-2019-106759 (PMC7691806; doi:10.1136/jmedgenet-2019-106759)
Supplement: Supplementary data [file jmedgenet-2019-106759supp002.pdf]

Appendix 2: Example of CanVIG-UK classification summary report

<table>
<tr><td colspan="6">Variant classification by Can-VIG UK (Cancer Variant Interpretation UK)
Can-VIG UK is a working group convened on behalf of the UK ACGS (Association of Clinical Genetic Scientists), which includes registered clinical scientists and clinical geneticists representing the following UK Regional molecular diagnostic laboratories: Aberdeen, Belfast, Birmingham, Bristol, Cambridge, Cardiff, Dublin, Exeter, Glasgow, GOSH, Guy's, Leeds, Liverpool (Cheshire & Merseyside), Oxford, Manchester, Newcastle, Nottingham, Sheffield , SW Thames (St George's), Wessex (Salisbury/Southampton), University Hospitals of Leicester.</td></tr>
<tr><td>Submitter</td><td colspan="3">Dr Clare Turnbull MD PhD FRCP FRCPath</td><td>Date</td><td>15/11/19</td></tr>
<tr><td>Gene</td><td>BRCA1</td><td>Transcript</td><td>NM_007294.3
ENST00000357654
LRG_292t1</td><td>Variant</td><td>c.53T>C (p.Met18Thr)</td></tr>
<tr><td>Population data</td><td colspan="5">The variant was observed in 7 independent UK families undergoing clinical diagnostic testing, the denominator of which dataset of clinical testing was 25,773. Case control comparison against ethnically matched population data (7/25,773 in familial cases against 0/64,603 GNOMAD NFE controls p_{exact}= 0.0015

There are additional reports of this variant in ClinVar (6), BIC (3) LOVD3 (22), UMD(7), DMuDB(7)

The variant is absent in the remainder of the GNOMAD populations (76,853 individuals)</td></tr>
<tr><td>Prediction (based on variant type/location), IN silico tools</td><td colspan="5">AlignGVGD class: C45
SIFT prediction: deleterious
MAPP prediction: bad
Polyphen2 HumVar prediction: benign
CADD scaled score [0-99]: 16.18
SuSPect score [0-100]: 95</td></tr>
<tr><td>Functional data</td><td colspan="5">Findlay et al. 2018: Non-functional via saturation editing analysis using haploid BRCA1 construct</td></tr>
<tr><td>Segregation data</td><td colspan="5"></td></tr>
<tr><td>De novo data</td><td colspan="5"></td></tr>
<tr><td>Allelic data (biallelic observations)</td><td colspan="5"></td></tr>
<tr><td>Other classifications</td><td colspan="5">Ambry LP 2018, Gene Dx LP 2018, Counsyl LP 2018 Color LP 2018
Enigma 2019 (multifactorial analysis): pathogenic</td></tr>
<tr><td>Other</td><td colspan="5"></td></tr>
</table>

**PATHOGENIC Criteria**	**Weight (supporting, moderate, strong, very strong)**	**BENIGN Criteria**	**Weight (supporting, strong)**
PVS1(null)		BS1/BA1 (controls)	
PS4 (case control)	Very strong	BP4 (in silico)	
PM2 (absent control)	Mod	BP1 (only trunc)	
PP3 (in silico)		BP7 (synonymous)	
PM5 (same residue)		BP3 (in frame, no func)	
PM1 (hot spot)		BS3 (functional assay)	
PP1 (Segregation)		BS4 (non segregation)	
PS3 (functional assay)	Strong	BP2 (biallelic)	
PM3 (biallelic)		BP6 (other databases)	
PP5 (other databases)	Sup	Alternative cause (BP5)	
Specific phenotype (PP4)			
De novo (PM6, PS2)			
**Total**	1 very strong, 1 strong, 1 mod, 1 sup	**Total**	
**Classification**	5-Pathogenic		

Appendix 3: CanVIG-UK Consensus Guidance for Variants of Reduced Penetrance in High Penetrance Cancer Susceptibility Genes

- Variant interpretation and classification should be undertaken using the ACMG framework (with ACGS and CanVIG-UK specifications)
- If any of the below criteria are met, the variant should be assigned the relevant ACMG class but with addendum of "reduced penetrance"
- The report should reference and recommend the nationally ratified clinical management recommendations for that gene for variants of reduced penetrance
- Clinical management recommendations for variants of reduced penetrance for each gene should be established by disease-specific experts

Criterion 1: Down-modification of classic biallelic phenotype

Abnormal physical AND cellular phenotype associated with biallelic mutations is present but notably milder

Example: BRCA2-related Fanconi anaemia:

- Cancer is not penetrant by 5 years **AND**
- Congenital abnormalities and physical features are mild **AND**
- Incomplete functional abrogation of chromosomal breakage following mitomycin C exposure **OR** BRCA2-specific assays show only modest depletion of BRCA2 in quantity and/or function

Criterion 2: Well calibrated assay gives intermediate effect

- Highly predictive and well-calibrated published functional assay demonstrate intermediate effect, ie significant impairment of protein function but not at level demonstrated for truncating mutations in gene (e.g. Guidugli et al for BRCA2[13 14], Findlay et al 2019 for BRCA1[15])

Criterion 3: Segregation analysis gives lower estimate of penetrance

- Formal genetic epidemiologic analyses demonstrate variant to be associated with disease but of penetrance statistically significantly reduced compared to established estimates eg: BRCA1 c.5096G>A p.Arg1699Gln[16 17]

12. ClinGen CDH1 Expert Panel Specifications to the ACMG/AMP Variant Interpretation Guidelines Version 2, 2019.
13. Guidugli L, Carreira A, Caputo SM, Ehlen A, Galli A, Monteiro AN, Neuhausen SL, Hansen TV, Couch FJ, Vreeswijk MP. Functional assays for analysis of variants of uncertain significance in BRCA2. *Human mutation* 2014;35(2):151-64. doi: 10.1002/humu.22478 [published Online First: 2013/12/11]
14. Guidugli L, Shimelis H, Masica DL, Pankratz VS, Lipton GB, Singh N, Hu C, Monteiro ANA, Lindor NM, Goldgar DE, Karchin R, Iversen ES, Couch FJ. Assessment of the Clinical Relevance of BRCA2 Missense Variants by Functional and Computational Approaches. *American journal of human genetics* 2018;102(2):233-48. doi: 10.1016/j.ajhg.2017.12.013 [published Online First: 2018/02/06]
15. Findlay GM, Daza RM, Martin B, Zhang MD, Leith AP, Gasperini M, Janizek JD, Huang X, Starita LM, Shendure J. Accurate functional classification of thousands of BRCA1 variants with saturation genome editing. *bioRxiv* 2018
16. Spurdle AB, Whiley PJ, Thompson B, Feng B, Healey S, Brown MA, Pettigrew C, Van Asperen CJ, Ausems MG, Kattentidt-Mouravieva AA, van den Ouweland AM, Lindblom A, Pigg MH, Schmutzler RK, Engel C, Meindl A, Caputo S, Sinilnikova OM, Lidereau R, Couch FJ, Guidugli L, Hansen T, Thomassen M, Eccles DM, Tucker K, Benitez J, Domchek SM, Toland AE, Van Rensburg EJ, Wappenschmidt B, Borg A, Vreeswijk MP, Goldgar DE. BRCA1 R1699Q variant displaying ambiguous functional

abrogation confers intermediate breast and ovarian cancer risk. *Journal of medical genetics* 2012;49(8):525-32. doi: 10.1136/jmedgenet-2012-101037 [published Online First: 2012/08/15]

17. Moghadasi S, Meeks HD, Vreeswijk MP, Janssen LA, Borg A, Ehrencrona H, Paulsson-Karlsson Y, Wappenschmidt B, Engel C, Gehrig A, Arnold N, Hansen TVO, Thomassen M, Jensen UB, Kruse TA, Ejlertsen B, Gerdes AM, Pedersen IS, Caputo SM, Couch F, Hallberg EJ, van den Ouweland AM, Collee MJ, Teugels E, Adank MA, van der Luijt RB, Mensenkamp AR, Oosterwijk JC, Blok MJ, Janin N, Claes KB, Tucker K, Viassolo V, Toland AE, Eccles DE, Devilee P, Van Asperen CJ, Spurdle AB, Goldgar DE, Garcia EG. The BRCA1 c. 5096G>A p.Arg1699Gln (R1699Q) intermediate risk variant: breast and ovarian cancer risk estimation and recommendations for clinical management from the ENIGMA consortium. *Journal of medical genetics* 2018;55(1):15-20. doi: 10.1136/jmedgenet-2017-104560 [published Online First: 2017/05/12]

Supplementary table 1: Variants reclassified to date in CanVIG-UK multidisciplinary meeting; RP: reduced penetrance

Date of CanVIG-UK review	Gene	Variant	Previous UK clinical classifications: most common (others)	CanVIG-UK consensus classification	Submission to ClinVar
Nov-17	BRCA1	c.53T>C p.Met18Thr	4 (3,5)	5	Y
Dec-17	BRCA2	c.8063T>C p.Leu2688Pro	3 (4,5)	5	Y
Jan-18	BRCA1	c.4096+3A>G	3 (2, 4)	2	Y
Feb-18	BRCA1	c.442-22_442-13del	3 (4,5)	4	Y
Mar-18	MLH1	c.122A>G p.Asp41Gly	3 (4,5)	5	Y
April-18	TP53	c.998G>A p.Arg333His	3 (4)	3	Y
Jun-18	BRCA2	c.9302T>G p.Leu3101Arg	4 (3,5)	5	Y
Jul-18	BRCA1	c.4963T>C p.Ser1655Pro	4 (3,5)	5	Y
Jul-18	BRCA1	c.4964C>T p.Ser1655Phe	4 (3,5)	5	Y
Jul-18	BRCA1	c.5207T>C p.Val1736Ala	3 (4)	5	Y
Aug-18	BRCA1	c.4357+6T>C	3 (4,5)	5	Y
Aug-18	BRCA2	c.8378G>A p.Gly2793Glu	3 (4,5)	5	Y
Sep-18	BRCA2	c.8524C>T p.Arg2842Cys	3 (4,5)	5 RP	Y
Oct-18	MLH1	c.1676T>G p.Leu559Arg	3 (4,5)	4	Y
Nov-18	MSH2	c.1807G>A p.Asp603Asn	4 (3,5)	4	Y
Jan-19	MLH1	c.440G>T p.Gly147Val	3 (4)	3	Y
Mar-19	MLH1	c.794G>A p.Arg265His	3	3	Y
Apr-19	BRCA1	c.5357T>C p.Leu1786Pro	3 (4)	4	Y
Apr-19	MLH1	c.1595G>A p.Gly532Asp	3 (4,5)	4	Y
May-19	APC	c.2497A>C p.Ser833Arg	3 (2)	3	Y
May-19	APC	c.6724A>G p.Ser2242Gly	3 (2)	2	Y
May-19	BMPR1A	c.1328G>A p.Arg443His	3 (4,5)	4	Y
May-19	BRCA1	c.5153-26 A>G	3 (4,5)	3	Y
May-19	BRCA2	c.520C>T p.Arg174Cys	3 (4)	3	Y
Sep-19	TP53	c.799C>T p.Arg267Trp	3 (4,5)	4	Y
Oct-19	TP53	c.1141A>G p.Lys381Glu	3 (4)	3	Y
Oct-19	TP53	c.322G>C p.Gly108Arg	4 (3,5)	3	Y
Oct-19	TP53	c.472 C>T p.Arg158Cys	3	3	Y
Oct-19	TP53	c.794T>A p.Leu265Gln	3 (4,5)	4	Y
Oct-19	TP53	c.560-14_560-13delCT	3 (4,5)	3	Y
Jan-20	TP53	c.453-455delCCC p.Pro153del	3 (4,5)	3	Y
